# Efficacy of COVID-19 treatments among geriatric patients: a systematic review

**DOI:** 10.1177/20499361221095666

**Published:** 2022-06-01

**Authors:** Helen Senderovich, Danusha Vinoraj, Madeline Stever, Sarah Waicus

**Affiliations:** Baycrest, 3560 Bathurst Street, Toronto, ON M6A 2E1, Canada; Department of Family and Community Medicine, University of Toronto, Toronto, ON, Canada; Division of Palliative Care, University of Toronto, Toronto, ON, Canada; Department of Psychiatry, Faculty of Medicine, University of Ottawa, Ottawa, ON, Canada; University of Limerick, Limerick, Ireland; School of Medicine, Trinity College Dublin, The University of Dublin, Dublin, Ireland

**Keywords:** clinical decisions, COVID-19, pharmacology, respiratory conditions, supportive care, symptoms and symptom management

## Abstract

**Introduction:**

A majority of the fatalities due to COVID-19 have been observed in those over the age of 60. There is no approved and universally accepted treatment for geriatric patients. The aim of this review is to assess the current literature on efficacy of COVID-19 treatments in geriatric populations.

**Methods:**

A systematic review search was conducted in PubMed, MedRxiv, and JAMA databases with the keywords COVID-19, geriatric, hydroxychloroquine, dexamethasone, budesonide, remdesivir, favipiravir, ritonavir, molnupiravir, tocilizumab, bamlanivimab, baricitinib, sotrovimab, fluvoxamine, convalescent plasma, prone position, or anticoagulation. Articles published from January 2019 to January 2022 with a population greater than or equal to 60 years of age were included. Interventions examined included hydroxychloroquine, remdesivir, favipiravir, dexamethasone, budesonide, tocilizumab, bamlanivimab, baricitinib, sotrovimab, convalescent plasma, prone position, and anticoagulation therapy. Outcome measures included viral load, viral markers, ventilator-free days, or clinical improvement.

**Results:**

The search revealed 302 articles, 52 met inclusion criteria. Hydroxychloroquine, dexamethasone, and remdesivir revealed greater side effects or inefficiency in geriatric patients with COVID-19. Favipiravir, bamlanivimab, baricitinib, and supportive therapy showed a decrease in viral load and improvement of clinical symptoms. There is conflicting evidence with tocilizumab, convalescent plasma, and anticoagulant therapy in reducing mortality, ventilator-free days, and clinical improvements. In addition, there was limited evidence and lack of data due to ongoing trials for treatments with sotrovimab and budesonide.

**Conclusion:**

No agent is known to be effective for preventing COVID-19 after exposure to the virus. Further research is needed to ensure safety and efficacy of each of the reviewed interventions for older adults.

## Introduction

**Figure 1.**
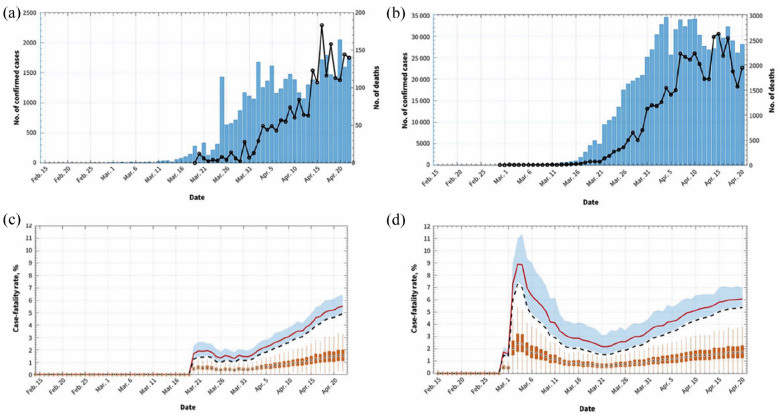
Daily incidence of COVID-19 (bars) and associated deaths (black line) in Canada (a) and the United States (b), 2020. Crude fatality rate (dashed line) and adjusted case fatality rate estimates (red line) from 19 March to 22 April 2020 in Canada (C) and the United States (D). Shaded area around red line shows 95% credible interval for the adjusted cast fatality ratio. Source: Abdollahi *et al.*^3^

On 11 March 2020, the World Health Organization (WHO) declared the viral infection caused by COVID-19 outbreak a global pandemic.^1^ Estimates of the case fatality rate vary during different times of the COVID-19 pandemic.^2,3^ Based on Canadian data from 22 April 2020, the crude mortality rate was reported at 4.9% which is noted to be variable between countries (Figure 1) depending on the availability of diagnostic testing and the capacity of national health care systems.^3^ The majority of deaths were seen in adults greater than 60 years old.^2,3^ Estimated case fatality ratio for COVID-19 patients stratified by age illustrate the detrimental effect of the virus on this population. Case data from China demonstrated that the case fatality ratio for patients less than 60 years old was estimated at 0.3%, while the estimated fatality ratio in patients of 60 years and older was 6.4%, and in patients of 80 years and older, the ratio was 13.4%.^4^ Increased risk of mortality in older patients has been seen in those who have symptoms of dyspnea, cardiovascular, cerebrovascular, and chronic obstructive pulmonary diseases.^5^ Increased heart, respiratory rate; prothrombin time; and leukocytopenia have been shown to be indicators of poor outcomes for older patients infected with COVID-19.^5^

Supportive care using antipyretics, opioids, antimicrobial agents, and oxygen are proposed in treatment protocols; however, the symptoms management between countries differ slightly.^6^ Research into various treatment protocols, including hydrochloroquine (HCQ), tocilizumab (TCZ), chloroquine phosphate, remdesivir (RMD), ritonavir (RTV), favipiravir (FVP), corticosteroids, and ivermectin have been proposed.^6^ In addition, passive immunization therapy with immunoglobulins has been suggested to reduce symptoms and mortality in patients with COVID-19 due to its effectiveness in previous epidemics and pandemics.^7,8^ Furthermore, supportive prone position (PP) or lateral position (LP) for patients with acute respiratory distress syndrome (ARDS) has shown to improve oxygenation and decrease mortality.^9^ Given the continuously evolving context of the COVID-19 pandemic, this systematic review was launched. The objective of this review is to assess the current knowledge of the COVID-19 treatment modalities for the geriatric population.

## Methods

A systematic review protocol was developed based on PRISMA guidelines. Articles for review were selected from electronic databases of PubMed, MedRxiv, and JAMA. Articles published between January 2019 and January 2022 that were in English or translated to English were included. Search terms included ‘COVID’ AND ‘geriatric’ AND (‘hydroxychloroquine’ OR ‘dexamethasone’ OR ‘budesonide’ OR ‘remdesivir’ OR ‘favipiravir’ OR ‘ritonavir’, ‘molnupiravir’ OR ‘tocilizumab’ OR ‘bamlanivimab’, ‘baricitinib’, ‘sotrovimab’, ‘fluvoxamine’ OR ‘convalescent plasma’ OR ‘prone position’ OR ‘anticoagulant’ OR ‘direct oral anticoagulant’ OR ‘heparin’ OR ‘antithrombotic’).

Geriatric population was defined as greater than or equal to 60 years of age. Observational cohort study (OCS) was defined as a clinically diagnosed group of infected patients who were treated with a specific treatment regimen (defined by exclusion criteria) and tested for viral markers at endpoint of treatment protocol. Distinct observational study (DOS) was defined as providing the same intervention to individual patients (distinct from OCS where articles reported results for the entire group) using an established and documented protocol and then, tested for viral markers at the end of the treatment. Randomized control trials (RCTs) were defined as studies that had two or more distinct groups of patients (treatment and control) and a clear treatment protocol. These patients were monitored at the end of the study for viral load, other viral markers, or clinical improvement.

*In vitro* study, animal study, case-control study, or case reports of a single individual, or of individuals who did not receive the same treatment protocol, other systematic reviews, literature reviews, and meta-analysis studies that did not involve the use of HCQ, RMD, FVP, RTV, molnupiravir (MPV), dexamethasone (DMS), budesonide (BUD), TCZ, bamlanivimab (BMB), baricitinib (BNB), sotrovimab (SVB), fluvoxamine (FLV), convalescent plasma (CP), PP, anticoagulant therapy, or no age data provided were excluded.

Inclusion criteria consisted of established treatment protocols using HCQ, RMD, FVP, RTV, MPV, DMS, BUD, TCZ, BMB, BNB, SVB, FLV, CP, PP, or anticoagulant therapy in older patients with demographic information expressed in the form of at least one of the following: age, age range, mean age ± SD, or median age ± SD. Studies that included particpants aged ⩾ 60 years old ± 1 SD of the mean or median that were statistically significant were included. Study designs consisting of either; RCT, OCS, or DOS and primary outcomes of one of the following: the improvement of COVID-19 symptoms/clinical improvement, shortened duration of COVID-19 symptoms, decreased viral load, and inflammatory markers such as C-reactive protein (CRP) and interleukin-6 (IL-6) were included.

Article titles were assessed based on exclusion criteria. Articles that passed the first assessment based on title exclusion had their abstracts reviewed. Entire articles were assessed for the remaining studies that met inclusion criteria. Studies that were not statistically significant were excluded from the review. Only studies that did not include any exclusion criteria and met all inclusion criteria were used in the systematic review analysis. Duplicate articles were removed prior to systematic review analysis. The level of evidence for the included studies was assessed using the Level of Evidence for Therapeutic Studies grading system.

### Patient and public involvement

Patients were not directly involved in this systematic review. All patient consent was given for the individual research articles examined.

## Results

**Figure 2.**
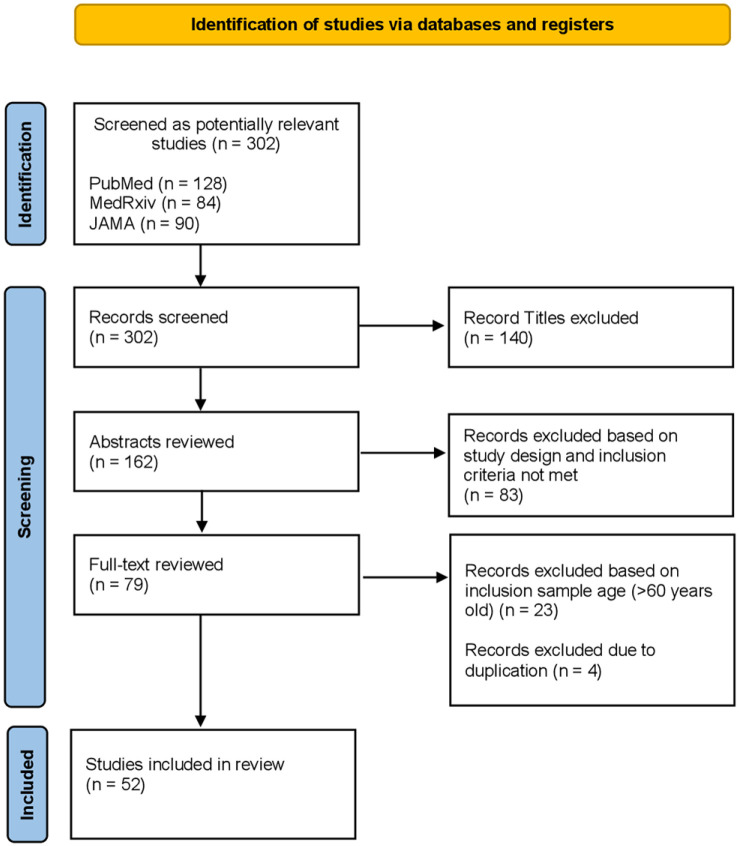
PRISMA flowchart.

As of January 2022, the search identified 302 potentially relevant studies. After review of these studies based on inclusion and exclusion criteria, 52 studies were eligible and included in the systematic review (Figure 2).

There were RCTs (*n* = 24), OCS (*n* = 21), and DOS (*n* = 7). Fourteen studies used HCQ (6 positive, 8 negative), 9 studies used CP (6 positive, 3 negative) as a treatment approach, 5 studies used RMD (2 positive, 3 negative), 1 study used BUD (1 positive), 2 studies of each used DMS (1 positive, 1 negative), 4 studies used TCZ (2 positive, 2 negative), 1 study used BMB (1 positive), 5 studies used BNB (4 positive, 1 negative), 1 study used SVB (1 positive), 3 studies used FVP (2 positive 1 negative), 3 studies used supportive PP (3 positive), and 5 studies used anticoagulation therapy (2 positive, 3 negative) (Table 1). The total number of patients reported in these studies were 30,265 (HCQ *n* = 9005; RMD *n* = 1749; BUD *n* = 4663; FVP *n* = 350; DMS *n* = 2062; TCZ *n* = 6764; BMB *n* = 1217; SVB = 583; CP *n* = 988; supportive PP *n* = 64; anticoagulation *n* = 2821). For the studies that listed patient demographic information as an age range, the widest age range was 22–97 years old. The narrowest age range was 67–71 years old.

**Table 1.** Overview of available evidence of analyzed studies.

Author [study identifier]	Study design	Intervention description	Demographic involved	Authors’ conclusions	CI/*p* value (if included in the study)	Level of evidence (for therapeutic studies)
Ahmad *et al.*^10^	DOS	Combination therapy of HCQ with doxycycline (100 mg po bid for 7 days). HCQ used in one of the following regimens: 200 mg po tid for 7 days, or 400 mg po bid on day 1 then 400 mg daily for the following 6 days.	54 patients from three different LTC facilities in New York City. Median age = 67 yo. Age range = 22–97 yo.	Clinical recovery (defined as resolution of fever, SOB, and/or return to clinical baseline) was seen in 46 patients (85% of treated patients).	N/A	4
Carlucci *et al.*^11^	OCS HCQ-AZT-Zinc treatment group (*n* = 411) HCQ-AZT treatment group (*n* = 521)	Combination therapy of HCQ-AZT (HCQ 400 mg followed by 200 mg bid daily for 5 days and AZT 500 mg once daily) either with or without zinc sulfate (220 mg capsule containing 50 mg elemental zinc po bid for 5 days).	932 patients from four acute care New York University Langone Health hospitals in New York City. Mean age HCQ-AZT-Zinc group = 63.2 ± 15.2 yo. Mean age HCQ-AZT group = 61.8 ± 16.0 yo	The addition of zinc to the HCQ-AZT therapy increased the frequency of patients being discharged, and improvement in respiratory function.	OR for hospital discharge in HCQ-AZT-zinc group compared with HCQ-AZT group = 1.53 (95% CI = 1.12–2.09). OR for reduction in mortality or transfer to hospice in the HCQ-AZT-zinc group compared with HCQ-AZT group = 0.4 (95% CI = 0.3–0.7)	2B
Chen *et al.*^12^	RCT Control group (*n* = 31) HCQ treatment group (*n* = 31) All received oxygen therapy, antiviral agents, antibacterial agents, and immunoglobulin.	HCQ group received oral HCQ sulfate tablets, 400 mg/day between days 1 and 5.	62 patients from Renmin Hospital of Wuhan University, China. Mean age = 44.7 ± 15.3 yo	HCQ could significantly shorten treatment and improve pneumonia.	Pneumonia was improved in 67% of all patients. 80.6% of patients treated with HCQ showed significant improvement of pneumonia.	2B
Gautret *et al.*^13^	RCT Control group (*n* = 16) Only HCQ treatment group (*n* = 14) HCQ and AZT treatment group (*n* = 6)	All patients were treated with oral HCQ sulfate 200 mg, tid for 10 days.	36 patients recruited from South France. Mean age = 45.1 ± 22 yo	HCQ was efficient in clearing CoV-19 within 3–6 days of treatment, assessed via NPS. Significant difference in viral load between HCQ groups and control beginning at 3 days post-starting treatment. Synergistic effect of the combination of HCQ and AZT. At 6 days into treatment, 100% of patients treated with HCQ and AZT were virologically cured.	Significant cure effect in patients with URTI and LRTI symptoms with respect to asymptomatic patients (*p* < 0.05).	1B
Gautret *et al.*^14^	OCS	200 mg of oral HCQ sulfate with AZT, tid for 10 days (500 mg on day 1 followed by 250 mg per day for the next 4 days)	80 patients from University Hospital Institute Méditerranée Infection in Marseille, France. Age range = 18–88 yo Median age = 52 yo	Virus cultures from respiratory samples were negative in 97.5% of patients at day 5. The HCQ and AZT treatment resulted in clinical improvement compared with outcomes of other hospitalized patients. All patients improved clinically except one 86-year-old patient who died.	Significant reduction in detectable viral RNA level (*R*^2^ = 0.9).	2B
Geleris *et al.*^15^	OCS 811 of 1376 patients received HCQ	HCQ 600 mg twice on day 1, then 400 mg daily for a median of 5 days.	1376 patients in large New York medical center. 832 patients aged 60 years or older.	HCQ use was not associated with either a lowered or increased risk of the end point of intubation or death. Findings do not support the use of HCQ outside RCT testing its efficacy.	HR for HCQ use and subsequent intubation or death = 1.04 (95% CI = 0.82–1.32).	2B
Mahévas *et al.*^16^	OCS 84 of 181 patients received HCQ treatment.	HCQ group received a dose of 600 mg/day within 48 hours of admission to hospital. Control group of standard care treatment without HCQ.	181 patient data collected at four tertiary care centers in France. Age range was 52–68 years.	HCQ seemed to have no effect on reducing admissions to intensive care or deaths at 21 days after admission. This study does not support the use of HCQ.	HR for survival in HCQ compared with control = 0.9 (95% CI = 0.4–2.1). Overall survival rate at day 21 post-treatment for HCQ group = 89% compared with control group = 91%.	2B
Molina *et al.*^17^	OCS	HCQ (600 mg/day for 10 days) and AZT (500 mg day 1 and 250 mg days 2–5)	11 patients Age range = 20–70 yo Mean age = 58.7 yo	Viral load in NPS remained positive for CoV-19 in 8 of the 10 patients. No evidence of clinical benefit of the treatment. No evidence of a strong antiviral activity or clinical benefit.	80% probability of remaining CoV-19 positive after treatment (95% CI = 49%–94%)	2B
Rosenberg *et al.*^18^	OCS	4 groups (i) HCQ + AZT (ii) HCQ alone (iii) Azithro-mycin alone (iv) Received neither drug.	1438 patient records reviewed from 25 hospitals in New York. Median age = 63 yo.	Treatment with HCQ, AZT, or both, compared with neither treatment was not significantly associated with differences in in-hospital mortality. Cardiac arrest was more frequent in patients who received HCQ with AZT compared with patients who received neither drug.	HR for HCQ and AZT = 1.35 (95% CI = 0.76–2.40). HR for HCQ = 1.08 (95% CI = 0.63–1.85). HR for AZT = 0.56 (95% CI = 0.26–1.21)	2B
Tang *et al.*^19^	RCT HCQ treatment group (*n* = 75). Standard-treatment group (*n* = 75).	Loading dose of 1200 mg daily for 3 days followed by a maintenance dose of 800 mg daily. Total treatment duration: 2–3 weeks.	150 patients in 16 treatment centers in Hubei, Henan, and Anhui provinces in China. Mean age HCQ group = 48.0 ± 14.1 yo Mean age control group = 44.1 ± 15 yo	No additional CoV-19 clearance through the use of adding HCQ to the current standard of care in patients with mainly persistent mild to moderate CoV-19.	Difference in viral clearance between the HCQ and control group = 4.1% (95% CI = −10.3% to 18.5%).	2B
Yu *et al.*^20^	OCS HCQ treatment group (*n* = 48) Standard-treatment group (*n* = 520)	200 mg of HCQ po bid for 7–10 days.	568 patients from Tongji Hospital in Wuhan, China. Median age = 68 yo There were 411 patients who were greater than 60 yo.	Use of HCQ was shown to significantly decrease mortality risk. Reduction in IL-6 levels from baseline measures after HCQ treatment but no change in the IL-6 levels from baseline after standard treatment.	HR for mortality in HCQ treatment group compared with standard treatment = 0.32 (95% CI = 0.16–0.62). *p* < 0.001. Significant reduction in IL-6 levels from baseline after HCQ treatment = 22.2 pg/ml. *p* = 0.002.	2B
Beigel *et al.*^21^	Double-blind RCT Placebo group (*n* = 521) RMD group (*n* = 538)	IV RMD (200 mg on day 1 followed by 100 mg on days 2–10) in single daily infusions.	1063 hospitalized patients from 73 hospitals in 10 countries (United States, United Kingdom, Denmark, Greece, Germany, Korea, Mexico, Spain, Japan, Singapore). Mean age RMD group = 58.6 ± 14.6 yo Mean age placebo group = 59.2 ± 15.4 yo	RMD was superior to placebo in shortening the time to recovery. Treatment with an antiviral drug alone is not likely to be sufficient. Risk for mortality was not significantly decreased with RMD compared with control.	Ratio rate to recovery for RMD to placebo group = 1.32 (95% CI = 1.12–1.55). *p* < 0.001. HR of mortality by 14 days for RMD to placebo group = 0.70 (95% CI = 0.47–1.04).	1B
Grein *et al.*^22^	OCS	Patients received IV RMD, 200 mg on day 1 followed by single daily infusions of 100 mg on days 2–10.	53 patients from Europe, North America, and Japan (United States: *n* = 22 Canada and Europe: *n* = 22 Japan: *n* = 9) Age range = 23–82 yo Median age = 64 yo	Clinical improvement was observed in 36 of the treated patients.	Clinical improvement in patients 70 years of age or older was less frequent than patients younger than 50 years (HR = 0.29, 95% CI = 0.11–0.74). Risk of death was greater in patients who were 70 years or older (HR = 11.34, 95% CI 1.36–94.17).	2B
Wang *et al.*^5^	Double-blind RCT Placebo group (*n* = 78) RMD group (*n* = 158)	IV RMD (200 mg on day 1 followed by 100 mg on days 2–10) in single daily infusions.	236 hospitalized patients from 10 hospitals in Hubei, China. Age range RMD treatment group = 57–73 yo Median age RMD group = 66 yo Age range Placebo group = 53–70 yo Median age Placebo group = 64 yo	Treatment of RMD was not associated with statistically significant clinical benefits.	HR for time to clinical improvement = 1.27 (95% CI 0.89–1.80)	1B
Ahn *et al.*^23^	DOS	Patients treated with 2 doses of 250 ml CP infusion administered at a 12-h interval between doses.	2 patients from Korea Aged: 71 yo and 67 yo	Both of the patient’s CRP and IL-6 levels returned to the normal range post-CP treatment.	N/A	4
Duan *et al.*^24^	OCS	A single dose 200 ml CP transfusion	10 patients from Wuhan Jinyintan Hospital, Jiangxia District Hospital of Integrative Traditional Chinese and Western Medicine, and First People’s Hospital of Jiangxia District in China. Age range = 34–78 yo Median age = 52.5 yo.	All patients achieved a negative viral load after CP infusion.	N/A	2B
Li *et al.*^25^	RCT Control group (*n* = 51) CP group (*n* = 52).	CP transfusion (4–13 ml/kg of recipient body weight). Administered at 10 ml for the first 15 min, then increased to 100 ml per hour with close monitoring.	103 patients from 7 medical centers in Wuhan, China. Median age = 70 yo.	No significant difference in time to clinical improvement between CP treatment group compared with control group within 28 days.	Difference in clinical improvement between CP and control group = 8.8% (95% CI = −10.4% to 28%). HR for CP to control group = 1.40 (95% CI = 0.79–2.49).	2B
Liu *et al.*^26^	OCS	2 units of 250 ml CP transfusion infused over 1–2 h.	39 patients from Mount Sinai Hospital, New York City. Mean age = 55 ± 13 yo.	Clinical condition in terms of oxygenation worsened in 18% of CP patients compared with 24% in matched controls. Improved survival rates for CP patients compared with matched controls.	Cochran–Mantel–Haenszel test for oxygenation between CP patients and matched controls: *p* = 0.167 HR for CP to control group = 0.2 (95% CI = 0.05–0.72). *p* = 0.02	2B
Salazar *et al.*^27^	DOS	Single 300 ml transfusion of CP treatment.	25 patients from Houston Methodist hospitals. Age range = 19–77 yo. Median age = 51 yo.	Clinical improvement (defined by improvement in the modified 6-point WHO ordinal scale) from baseline assessment prior to treatment was seen in 9 patients (36%) at day 7 post-infusion. At day 14 post-infusion, 19 patients (76%) had a clinical improvement from baseline. Median value of CRP in transfused patients decreased with respect to time post-infusion. 14.7 mg/dl at day 0, 2.9 mg/dl at day 7 post-infusion, and 0.45 mg/dl at day 14 post-infusion.	N/A	4
Shen *et al.*^28^	DOS	CP treatment between 10 and 22 days after their hospital admission. CP treatment protocol consisted of two consecutive transfusions of 200 ml (400 ml total) on the same day it was obtained from the donor.	5 patients from Shenzhen Third People’s Hospital, China. Age range = 36–65 yo.	Clinical status of all patients improved following CP treatment. Neutralizing antibody titers were increased after CP transfusion for up to 7 days post transfusion.	N/A	4
Ye *et al.*^29^	DOS	CP transfusion (200 ml/cycle) for 30 min. Transfusion protocol in accordance to New Coronavirus Pneumonia CP Therapy Guidance of China (2nd edition).	6 patients admitted to Wuhan Huoshenshan Hospital, China. Age range = 28–75 yo.	3 patients had a decrease in anti-CoV-19 IgM and IgG antibodies after CP therapy and 4 patients had a negative throat swab viral load test after CP therapy. There was clinical benefit in all 6 patients treated with CP	N/A	4
Dong *et al.*^30^	DOS	Patients provided with a daily PP session. Actual hours of tolerated PP were self-reported by patients. For patients who did not tolerate the PP, a combination of PP with LP, or LP only was provided.	25 patients admitted to Renmin Hospital of Wuhan University. Mean age = 54.4 ± 16.1 yo	Mean respiratory rate for all patients decreased from 28.4 ± 3.5 breaths/min to 21.3 ± 1.3 breaths/min after various durations of PP or LP sessions. Assessment of all patient’s chest CT showed improvement.	N/A	4
Elharrar *et al.*^31^	OCS	ABG were measured just prior to PP, during PP, and 6–12 h after resupination.	24 patients from a single health center in France who were awake, non-intubated, spontaneously breathing. Mean age = 66.1 ± 10.2 yo.	63% of patients were able to tolerate PP for more than 3 h. However, oxygenation increased during PP in only 25% of patients and was not sustained in half of those patients after resupination.	In patients who could withstand PP for more than 3 h, there was an increase in PaO2 by 21.3 mmHg (95% CI = 6.3–36.3). No significant difference between PaO2 before PP and after resupination (*p* = 0.53).	2B
Sartini *et al.*^32^	DOS	Respiratory rate, PaO2, FIO2, and O2 sat were reported after using NIV in the PP.	15 patients from a single health center in Italy who received NIV in the PP in 2 cycles for a total duration of 3 h. Mean age = 59 ± 6.5 yo.	Compared with baseline, all patients had a reduction in respiration rate during and after pronation. All patients have an improvement in O2 sat and PaO2: FIO2 during PP.	Respiratory rate reduction (*p* < 0.001). Improvement in O2 sat and PaO2: FIO2 during PP (0.001).	2B
Cai *et al.*^33^	Unrandomized, open-label trial 35 patients in FVP group; 45 patients in LPV/RTV group (control).	2 groups: Oral FVP (day 1 = 1600 mg bid po; days 2–14 = 600 mg bid po). LPV/RTV (days 1–14 = 400 mg/100 mg bid po). Both groups also received INF-α by aerosol inhalation (5 million IU bid).	80 patients from Third People’s Hospital of Shenzhen, China. Median age = 47 yo. Number of patients greater than or equal to 65 yo = 11.	Patients treated with FVP had a statistically significant decrease in treatment time to viral clearance and significant improvement in chest imaging compared with control.	Time to viral clearance: FVP = 4 days; LPV/RTV = 11 days (*p* < 0.001). Improvement in chest imaging: FVP = 91.43% improvement; LPV/RTV = 62.22% (*p* < 0.005).	1B
Chen *et al.*^34^	Multi-centered RCT. 116 patients in FVP group. 120 patients in arbidol group (control).	1:1 ratio of patient randomization to FVP and control (4 patients lost in FVP group from analysis). FVP group: day 1 = 1600 mg bid po, days 2 to experimental endpoint (days 7–10) = 600 mg bid po. Control group: day 1 to experimental endpoint (days 7–10) = arbidol 200 mg tid.	240 patients from Zhongnan hospital of Wuhan University (*n* = 120), Leishenshan Hospital (*n* = 88), Third People’s Hospital of Hubei Province (*n* = 32). Number of patients greater than or equal to 65 yo = 70.	In previously untreated patients, FVP had a higher clinical recovery rate (recovery of fever, respiratory rate abnormalities/instability, oxygen saturation, cough).	Clinical recovery rate in FVP group *versus* control = 71.43% *versus* 55.86% (*p* < 0.05).	1B
Lou *et al.*^35^	RCT	1:1:1 ratio of patient randomization to FVP, BLMB, control groups. BLMB group: days 1 and 4 = 80 mg po. For virally positive patients at day 7, they were given another 80 mg po. FVP group: days 1–14 = loading dose of 1600 mg or 220 mg po, followed by 600 mg tid po. Control group: 100,000 IU inhaled INF-α tid or qid with either LPV/RTV (400 mg/100 mg bid po) or DR/CB (800 mg/150 mg qid po) with arbidol (200 mg tid or qid).	30 patients from The First Affiliated Hospital, Zhejiang University School of Medicine. Mean age = 52.5 ± 12.5 yo.	Adding BLMB or FVP to current treatment does not provide additional benefits to clinical outcome in the treatment for CoV-19. The percentage of patients who showed viral clearance at day 14 of the treatment protocol was 70%, 77%, and 100% in BLMB, FVP, and control groups, respectively.	N/A	2B
Wu *et al.*^36^	Retrospective OCS	Treatment group: received corticosteroid treatment. Variable dosing and duration.	1763 patients (severe patients *n* = 1514; critical patients *n* = 249) from Wuhan Hankou Hospital and No. Six Hospital of Wuhan, China. Severe patients: Median age = 61 yo (IQR = 51–70 yo). Critical patients: Median age = 68 yo (IQR = 58–78 yo).	In-hospital mortality rate was significantly higher in the corticosteroid group compared with the control in severe and critical cases of CoV-19.	HR for mortality in treatment group compared with control: Severe cases = 1.77 (95% CI = 1.08–2.89; *p* < 0.05). Critical cases = 2.07 (95% CI = 1.08–3.98; *p* < 0.05).	2B
Tomazini *et al.*^37^	RCT	20 mg of DMS intravenously daily for 5 days, 10 mg of DMS daily for 5 days or until ICU discharge, plus standard care (*n* = 151) or standard care alone (*n* = 148).	299 patients, 151 received intervention and standard of care, 148 received standard of care alone Median age of intervention group 60.1 (SD = 15.8)	Among patients with COVID-19 and moderate or severe ARDS, use of intravenous DMS plus standard care compared with standard care alone resulted in a statistically significant increase in the number of ventilator-free days (days alive and free of mechanical ventilation) over 28 days.	Patients randomized to the DMS group had a mean 6.6 ventilator-free days (95% CI = 5.0–8.2) during the first 28 days *versus* 4.0 ventilator-free days (95% CI = 2.9–5.4) in the standard care group (difference, 2.26; 95% CI = 0.2–4.38; *p* = .04). At 7 days, patients in the DMS group had a mean SOFA score of 6.1 (95% CI = 5.5–6.7) *versus* 7.5 (95% CI = 6.9–8.1) in the standard care group (difference, −1.16; 95% CI = −1.94 to −0.38; *p* = .004).	2B
Simonovich *et al.*^38^	Double-blind RCT	Hospitalized adult patients with severe COVID-19 pneumonia were randomized in a 2:1 ratio to receive CP or placebo	334 patient, 228 patients received CP, 105 received placebo Median age in those receiving CP 62.5	No significant differences were observed in clinical status or overall mortality between patients treated with CP and those who received placebo.	At day 30, no significant difference was noted between the CP group and the placebo group in the distribution of clinical outcomes according to the ordinal scale (odds ratio, 0.83 (95% CI = 0.52–1.35; *p* = 0.46). Overall mortality was 10.96% in the CP group and 11.43% in the placebo group, for a risk difference of −0.46 percentage points (95% CI = −7.8 to 6.8).	1B
Mitja *et al.*^39^	Cluster RCT	HCQ group (received the drug at a dose of 800 mg once, followed by 400 mg daily for 6 days)	2314 healthy contacts of 672 index case patients with COVID-19. 1116 contacts were randomly assigned to receive HCQ and 1198 to receive usual care. Mean age = 48.6 ± 19.0 yo	Post-exposure therapy with HCQ did not prevent SARS-CoV-2 infection or symptomatic COVID-19 in healthy persons exposed to a PCR-positive case patient.	Results were similar in the HCQ and usual-care groups with respect to the incidence of PCR-confirmed, symptomatic COVID-19 (5.7% and 6.2%, respectively; risk ratio, 0.86 (95% CI = 0.52–1.42). In addition, HCQ was not associated with a lower incidence of SARS-CoV-2 transmission than usual care (18.7% and 17.8%, respectively).	2B
RECOVERY Collaborative Group^40^	RCT	Patients received HCQ sulfate (in the form of a 200-mg tablet containing a 155-mg base equivalent) in a loading dose of four tablets (total dose, 800 mg) at baseline and at 6 h, which was followed by two tablets (total dose, 400 mg) starting at 12 h after the initial dose and then every 12 h for the next 9 days or until discharge, whichever occurred earlier	1561 patients received HCQ and 3155 received usual care. Mean age of intervention group 65.2 ± 15.2 yo	Among patients hospitalized with COVID-19, those who received HCQ did not have a lower incidence of death at 28 days than those who received usual care.	Death within 28 days occurred in 421 patients (27.0%) in the HCQ group and in 790 (25.0%) in the usual-care group (rate ratio, 1.09; 95% CI = 0.97–1.23; *p* = 0.15).	2B
Self *et al.*^41^	Double-blind RCT	Patients were randomly assigned to HCQ (400 mg twice daily for 2 doses, then 200 mg twice daily for 8 doses) (*n* = 242) or placebo (*n* = 237).	Intervention group *n* = 242 and placebo *n* = 237. Median age = 57 (range = 44–68)	Among adults hospitalized with respiratory illness from COVID-19, treatment with HCQ, compared with placebo, did not significantly improve clinical status at day 14.	Clinical status on the ordinal outcome scale at 14 days did not significantly differ between the HCQ and placebo groups.	1B
Agarwal *et al.*^42^	RCT	Participants in the intervention arm received two doses of 200 ml CP, transfused 24 h apart.	464 adults (⩾18 years) admitted to hospital; 235 were assigned to CP with best standard of care (intervention arm) and 229 to best standard of care only (control arm). Median age = 51 (range: 41–60)	CP was not associated with a reduction in progression to severe COVID-19 or all-cause mortality.	Progression to severe disease or all-cause mortality at 28 days after enrolment occurred in 44 (19%) participants in the intervention arm and 41 (18%) in the control arm (risk difference 0.008 (95% CI = −0.062 to 0.078); risk ratio 1.04, 95% CI = 0.71–1.54).	2B
Goldman *et al.*^43^	RCT	Patients were randomly assigned in a 1:1 ratio to receive intravenous RMD for either 5 days or 10 days. All patients received 200 mg of RMD on day 1 and 100 mg once daily on subsequent days.	In total, 397 patients underwent randomization and began treatment (200 patients for 5 days and 197 for 10 days).	In patients with severe COVID-19 not requiring mechanical ventilation, our trial did not show a significant difference between a 5-day course and a 10-day course of RMD.	After adjustment for baseline clinical status, patients in the 10-day group had a distribution in clinical status at day 14 that was similar to that among patients in the 5-day group (*p* = 0.14).	2B
Gupta *et al.*^44^	Retrospective OCS	Critically ill adults with COVID-19 were categorized according to whether they received or did not receive TCZ in the first 2 days of admission to the ICU.	4485 adults with COVID-19 admitted to participating ICUs at 68 hospitals across the United States Median age = 62 (range: 52–71)	Risk of in-hospital mortality in this study was lower in patients treated with TCZ in the first 2 days of ICU admission compared with the control group.	Patients treated with TCZ had a lower risk of death compared with those not treated with TCZ (HR, 0.71; 95% CI = 0.56–0.92). The estimated 30-day mortality was 27.5% (95% CI = 21.2%–33.8%) in the TCZ-treated patients and 37.1% (95% CI = 35.5%–38.7%) in the non-TCZ-treated patients.	2B
Salvarani *et al.*^45^	RCT	Patients hospitalized with COVID-19 pneumonia randomized to receive TCZ or standard of care in 24 h. The experimental arm received TCZ intravenously within 8 h from randomization at a dose of 8 mg/kg up to a maximum of 800 mg, followed by a second dose after 12 h.	126 patients were randomized (60 to the TCZ group; 66 to the control group). Median age = 60 (range: 53–72)	Hospitalized adult patients with COVID-19 pneumonia and PaO2/FIO2 ratio between 200 and 300 mmHg who received TCZ had no benefit on disease progression compared with standard care.	17 patients of 60 (28.3%) in the TCZ arm and 17 of 63 (27.0%) in the standard care group showed clinical worsening within 14 days since randomization (rate ratio, 1.05; 95% CI = 0.59–1.86).	2B
Hermine *et al.*^46^	RCT	Patients were randomly assigned to receive TCZ, 8 mg/kg, intravenously plus usual care on day 1 and on day 3 if clinically indicated (TCZ group) or to receive usual care alone (UC). UC included antibiotic agents, antiviral agents, corticosteroids, vasopressor support, and anticoagulants.	Of 131 patients, 64 were randomly assigned to the TCZ group and 67 to UC group. Median age = 64 (range: 57.1–74.3) yo	Patients with COVID-19 and pneumonia requiring oxygen support but not admitted to the ICU, TCZ did not reduce WHO-Combined and Positive scores lower than 5 at day 4 but might have reduced the risk of non-invasive ventilation, mechanical ventilation, or death by day 14. No difference on day 28 mortality was found.	The HR for MV or death was 0.58 (90% CrI, 0.30–1.09). At day 28, 7 patients had died in the TCZ group and 8 in the UC group (adjusted HR, 0.92; 95% CI 0.33–2.53). Serious adverse events occurred in 20 (32%) patients in the TCZ group and 29 (43%) in the UC group (*p* = .21).	2B
Horby *et al.*^47^	RCT	Trial participants with hypoxia and evidence of systemic inflammation were eligible for randomization to alone *versus* UC plus TCZ at a dose of 400–800 mg (depending on weight) given intravenously. A second dose could be given 12–24 h later if the patient’s condition had not improved.	2022 patients were randomly allocated to TCZ and 2094 UC groups. Mean age = 63.6 (SD ± 13.7) yo	In hospitalized COVID-19 patients with hypoxia and systemic inflammation, TCZ improved survival and other clinical outcomes such as lower 28-day mortality such as regardless of the level of respiratory support and addition of systemic corticosteroids.	Allocation to TCZ was associated with a greater probability of discharge from hospital alive within 28 days (54% *versus* 47%; rate ratio 1.22, 95% CI = 1.12–1.34, *p* < 0.0001)	2B
Gottlieb *et al.*^48^	RCT	Trial participants who tested positive for COVID-19 had one or more mild to moderate symptom. Patients were randomized to receive a single infusion on BMB 700, 2800, or 7000 mg, combination treatment of BMB with etesevimab each 2800 mg or placebo.	613 patients were randomized to receive a single infusion on BMB 700 mg (*n* = 101), 2800 mg (*n* = 107), or 7000 mg (*n* = 101), combination treatment of BMB (2800 mg) with etesevimab (2800 mg) (*n* = 112), or placebo (*n* = 156). 69 patients were > 65 yo.	Among nonhospitalized patients, bamlanivimab and etesevimab, compared with placebo, was associated with a statistically significant reduction in SARS-CoV-2 viral load at day 11; no significant difference in viral load reduction was observed for bamlanivimab monotherapy.	Treatment with BMB and etesevimab compared with placebo was associated with a statistically significant (*p* = 0.01) viral load reduction at day 11; however, there was no significant viral load reduction using BMB monotherapy. Immediate hypersensitivity reactions were reported for 9 out of 613 patients with BMB monotherapy, and no deaths occurred during the treatment study.	2B
Abizanda *et al.*^49^	OCS	Patients hospitalized for moderate-to-severe COVID with confirmed pneumonia separated into age brackets < 70 yo, or > 70 yo.	328 patients were in the < 70 yo bracket (86 with BNB and 86 controls) or > 70 yo (78 on BNB and 78 control). Dosage was at 4 mg for 14 days. Mean age = 79.1 yo	Treatment with BNB resulted in significant reduction in death from any cause (48% > 70 yo) and 18.5% reduction in 30-day absolute mortality risk (20.8%).	Patients given BNB had a 18.5% reduction in 30-day absolute mortality risk (*n*/*N*: 16/78 (20.5%) 30/78 (38.5%) in matched controls, *p* < 0.001) and a lower 30-day adjusted fatality rate (HR 0.21; 95% CI = 0.09–0.47; *p* < 0.001).	2B
Stebbing *et al.*^50^	OCS	238 patients with confirmed COVID and severe pneumonia given 8 or 4 mg of BNB orally for 14 days during hospital stay.	122 patients in high-dose group receiving 8 mg BNB. 116 patients in usual-dose group received 4 mg for 14 days. Mean age = 63 (range: 54.8–69) yo	Blood oxygen saturation was stabilized earlier in high-dose group compared with low-dose group. High-dose group required less intensive unit intubation support compared with the usual-dose group. 30-day mortality and 60-day rehospitalization rate were higher in the usual-dose group than the high-dose group.	Blood oxygen saturation level was stabilized (⩾94% on room air) earlier in the high-dose group (5 (IQR: 4–5)/8 (IQR: 6–9), *p* < 0.05). Usual-dose group had more intensive care needs compared with high-dose group (17.2%/9%, *p* < 0.05; 11.2%/4.1%, *p* > 0.05; *N* = 116/122, respectively). 30-day mortality and 60-day rehospitalization rate were higher in the usual-dose group than the high-dose group (6%/3.3%, *p* < 0.01; 11.9%/7.6%, *p* > 0.05; *N* = 116/122).	2B
Bronte *et al.*^51^	OCS	An observational longitudinal study using BNB in a treatment group of 20 patients for 14 days. A clinical onset of symptoms not exceeding 9 days and the presence of interstitial lung involvement not exceeding 50% on chest x-ray or CT were indicated to receive BNB.	20 patients were treated with off-label use of BNB. 4 mg of BNB twice daily for 2 days, followed by 4 mg per day for the remaining 7 days. The non-BNB group received HCQ or antiviral therapy RTV. Mean age = 68 yo (range: 60.5–78.5 yo)	Patients treated with BNB had marked reduction of serum IL-6, IL-1B and TNF-alpha, rapid recovery of circulating T and B cell frequencies, and increased antibody production against the spike protein. Clinical association with reduction in need for oxygen therapy noted.	1 death in the BNB group compared with 25 deaths in the non-BNB group who received HCQ or RTV (*p* < 0.001). There were 3 ARDS events compared with 15 in HCQ/RTV group (*p* = 0.37). Duration of hospitalization was similar, 12 days compared with 11 days in the HCQ/RTV group (*p* = 0.28).	2B
Rosas *et al.*^52^	OCS	Retrospective review of medical records of patients with confirmed COVID and pneumonia with a PaO2/FiO2 < 300 treated with either BNB or TCZ.	60 patients were included, 23 patients received BNB monotherapy (2–4 mg daily), 31 received TCZ (IV dose 400 mg) as monotherapy and 11 patients received both BNB and TCZ. Mean age 67 yo (SD = 14)	No significant mortality benefits were found within either treatment groups and no side effects noted. The BNB monotherapy showed a reduction in temperature, CRP, D dimer, or increase in oxygen saturation requirement and decrease respiratory rate compared with TCZ. Combined group had worsened PaO2/FiO2 levels compared with monotherapies.	Patients receiving BNB monotherapy had a significant reduction in temperature, CRP, D dimer, or increase in oxygen saturation requirement and decrease respiratory rate compared with TCZ (*p* < 0.01). The combined group had PaO2/FiO2 average at discharge low, requiring intensive care intubation (27%).	2B
Izumo *et al.*^53^	OCS	Patients at Red Cross with severe COVID received triple therapy BNB (<14 days), RMD (<10 days), DMS (<10 days).	44 patients with severe COVID were enrolled all received combination therapy of BNB (4 mg daily dose orally or through NG tube for 14 days), RMD (IV 200 mg loading dose followed by 100 mg 10 days), and DMS (oral or IV 6mg daily for 10 days). Mean age 61 yo (range: 55–75 yo)	Patients who received BNB had median hospitalization of 11-day, time to recovery 9 days, duration of ICU stay 6 days, duration of ventilation 5 days, supplemental oxygen.	In combination triple therapy with BNB, there was an overall low mortality rate (2.3%) and decrease in requirement for invasive mechanical ventilation (90%). Incidence of adverse events 34% (15/44).	2B
Gupta *et al.*^54^	RCT	Multi-center double RCT phase 3 assigned symptomatic COVID patients to receive SVB or placebo. Patients needed to have one risk factor for disease progression, with less than 5 days after onset of symptoms.	Total of 583 patients, 193 were over 65 yo. 93 patients were randomized into the SBV (500 mg IV) group, 93 received placebo.	3 patients in the SVB group, compared with 21 patients in the placebo group had disease progression leading to hospitalization or death. In the placebo group, 5 patients were admitted to ICU with 1 death. Adverse events were reported by 17% of the patients in the SVB group and 19% in placebo group.	3 patients (1%) in the SVB group compared with 21 patients (7%) in the placebo group had disease progression leading to hospitalization or death. (Relative RR 85%; 97.24% CI, 44–96; *p* = 0.002)	1B
Yu *et al.*^55^	RCT	Multi-center, open-label RCT involved in individuals aged >65 or >50 yo with comorbidities and a history of being unwell <14 days due to suspected COVID. Participants were randomized to standard care, standard care plus inhaled BUD, or usual-care (antipyretics) group.	4663 participants with suspected COVID, 2617 tested positive. 751 were randomized with BUD (800 µg twice daily for 14 days), 1028 were given usual care (antipyretics) and 643 were given other interventions (antibiotics such as AZT). Mean age 62.8 yo (range: 50–100 yo)	Inhaled BUD reduced time to recovery by median of 3 days in individuals with COVID and comorbidities.	Self-reported recovery of an estimated 2–94 days in the BUD group *versus* the usual-care (antipyretic) group (11 to 8 days (95% BCI 10.0–14.1) *versus* 14 to 7 days (12.3–18.0); HR 1.21 (95% BCI 1.08–1.36) Hospital admission or death outcome estimated rate was 6%–8% (95% BCI 4.1–10·2) in the BUD group *versus* 8·8% (5.5–12.7) in the usual-care group (antipyretics) [estimated absolute difference 2·0% (95% BCI −0.2 to 4.5); OR 0·75 (95% BCI 0.55–1.03)] Two participants in the BUD group and four in the standard care (antipyretic) group had serious adverse events unrelated to COVID.	1B
Fumagalli *et al.*^56^	OCS	806 patients with COVID-19 aged >60 yo in a multi-center observational study (GeroCOV study) with comorbidity of atrial fibrillation who were on antiplatelet or oral anticoagulant therapy	806 patients enrolled in hospital settings who were taking either oral anticoagulants, antiplatelet agents, or no antithrombotic therapy. 51% of the patients were switched to low-molecular-weight heparins. Mean age: 78 ± 9 years	Oral anticoagulant use before and during hospitalization was higher among survivors. Lower age, higher self-sufficiency and less severe initial COVID-presentation, using vitamin K antagonists or DOAC at admission or hospitalization were associated with lower in-hospital death.	Patients with atrial fibrillation who survived were younger (81 ± 8 *versus* 84 ± 7 years; *p* = 0.002) and had a lower CHA2DS2-VASc score (3.9 ± 1.6 *versus* 4.4 ± 1.3; *p* = 0.02) than those who died. DOAC use before (63.1 *versus* 32.3%; *p* < 0.001) and during hospitalization (34.0 *versus* 12.7%; *p* = 0.002) was higher among survivors. At multivariable analysis, lower age, higher self-sufficiency, less severe initial COVID-19 presentation, and the use of vitamin K antagonists (odds ratio (OR) 95% confidence interval (CI) 0.03–0.84) or DOAC (OR = 0.22, 95% CI: 0.08–0.56) at admission, or the persistence of OAC during hospitalization (OR = 0.05, 95% CI: 0.01–0.24), were associated with a lower chance of in-hospital death.	2B
Rossi *et al.*^57^	Retrospective OCS	Older patients (>70 yo) with COVID-19 and interstitial pneumonia with comorbid cardiovascular diseases such as HTN, DM, CAD, CVA, CHF, PE, hypercholesterolemia, obesity, or valvulopathy.	70 patients > 70 yo with known CAD with a diagnosis of COVID-19 chronically (>6 mo) were followed taking aspirin (58.1%), P2Y12 inhibitors (12.9%), dual antiplatelet therapy (6.4%), DOAC therapy (22.6%), beta-blockers (48.4%), statins (38.7%), ACE (58.1%), ARB (29%), or calcium-antagonists (9.7%). Median age: 79 (range: 70–92 yo)	COVID-19 patients with CAD had worse prognosis, cardio-active treatment has protective role in COVID-19 pneumonia and anticoagulant chronic consumption reduced mortality.	Anticoagulant chronic use in the survivor group was high (48.7%; *p* < 0.001). Anticoagulant drugs, specifically chronic DOAC intake is associated with decreased mortality risk in the older population (HR 0.38 (0.17–0.58 95% CI), *p* < 0.01. Other cardio-active drugs did not induce mortality risk including ACE and ARB.	2B
Olcott *et al.*^58^	Retrospective OCS	Older patients (>70 yo) who were admitted in hospital with COVID-19 who were previously on anticoagulant therapy.	309 patients > 70 yo were on DOACs (22%) or warfarin (4%) compared with no anticoagulant (74%). Median age: 83 (range: 70–101)	There was no statistically significant improvement in all-cause mortality for patients who were anticoagulated preadmission.	Proportionally more male patients had died compared with females (*p* = 0.021; OR 1.47; 95% CI = 0.94–2.30; *p* = –.094). There was no statistically significant improvement in all-cause mortality for patients who were anticoagulated preadmission on univariate analysis (OR 0.95; 95% CI = 0.56–1.57; *p* > 0.05)	2B
Sadeghipour *et al.*^59^	RCT	Multi-center RCT of patients with confirmed COVID-19 admitted to the ICU randomized to the intermediate-treatment dose or continued standard-dose of prophylactic anticoagulation groups.	562 patients were randomized to receive intermediate-treatment dose (enoxaparin, 1 mg/kg once daily) (*n* = 276) *versus* standard prophylactic anticoagulation (enoxaparin, 40 mg daily) (*n* = 286) 30-day follow-up.	Prophylactic anticoagulation, compared with standard-treatment dose of anticoagulation did not result in a significant difference in venous or arterial thrombosis, or morality within 30 days.	Venous or arterial thrombosis occurred in 126 patients (45.7%) in the intermediate-treatment dose group and 126 patients (44.1%) in the standard-dose prophylaxis group [absolute risk difference, 1.5% (95% CI = −6.6% to 9.8%); OR, 1.06 (95% CI = 0.76–1.48); *p* = 0.70)]. Major bleeding occurred in 7 patients (2.5%) in the intermediate-treatment dose group and 4 patients (1.4%) in the standard-dose prophylaxis group (risk difference, 1.1% (one-sided 97.5% CI, −∞ to 3.4%); OR, 1.83 (one-sided 97.5% CI, 0.00–5.93). Severe thrombocytopenia occurred only in patients assigned to the intermediate-treatment dose group (6 *versus* 0 patients; risk difference, 2.2% (95% CI = 0.4%–3.8%); *p* = 0.01).	2B
Zarychanski *et al.*^60^	RCT	RCT of patients with severe COVID-19 with a requirement for organ support, high flow nasal cannula, non-invasive or invasive ventilation, vasopressors or inotropes given anticoagulant therapy or thromboprophylaxis in hospital.	1074 patients were randomized into therapeutic anticoagulation with unfractionated or low molecular weight heparin (*n* = 529), or thromboprophylaxis including DOAC (*n* = 545) Mean age: 60.2 (SD = 13.1) Therapeutic anticoagulation Mean age: 61.6 (SD 12.5) thromboprophylaxis	Patients with severe COVID-19, therapeutic anticoagulation did not improve hospital survival or days free of organ support compared with thromboprophylaxis.	Median organ support-free days were 3 days (IQR −1, 16) in patients assigned to therapeutic anticoagulation and 5 days (IQR −1, 16) in patients assigned to usual-care thromboprophylaxis (OR 0.87, 95% CrI 0.70–1.08, posterior probability of futility (OR < 1.2) 99.8%). Hospital survival was comparable between groups (64.3% *versus* 65.3%, adjusted odds ratio 0.88, 95% CrI 0.67–1.16). Major bleeding occurred in 3.1% of patients assigned to therapeutic anticoagulation and 2.4% of patients assigned to usual-care thromboprophylaxis.	2B

ACE, angiotensin converting enzymes; ARB, angiotensin II receptor blockers; ARDS, acute respiratory distress syndrome; AZT, azithromycin; BMB, bamlanivimab; BNB, baricitinib; BUD, budesonide; CAD, coronary heart disease; CHA2DS2-VASc, congestive heart failure, hypertension, age, diabetes, stroke/transient ischemic attack vascular score; CI, confidence interval; CP, convalescent plasma; CrI, credible interval; CRP, C-reactive protein; DMS, dexamethasone; DOAC, direct oral anticoagulants; DOS, distinct observational study; favipiravir; HCQ, hydrochloroquine; HR, hazard ratio; ICU, intensive care unit; IL-6, interleukin-6; IQR, interquartile range; LP, lateral position; *n*, number of individuals; OCS, observational cohort study; OR, odds radio; PP, prone position; RCT, randomized control trial; RMD, remdesivir; RTV, ritonavir; SD, standard deviation; SVB, sotrovimab; TCZ, tocilizumab; UC, usual care; WHO, World Health Organization; yo, years old; ABG, arterial blood gas; BCI, Bayesian credible interval; BLMB, baloxivir; CHF, chronic heart failure; CVA, cardiovascular accident; DM, diabetes mellitus; FVP, favipiravir; HTN, hypertension; LPV, lopinavir; LRTI, lower respiratory tract infection; LTC, long-term care; MV, mechanical ventilation; NG, nasogastric; NIV, non-invasive ventilation; NPS, national prescribing service; PE, pulmonary embolism; RR, respiratory rate; SBV, sotrovimab; SOB, shortness of breath; SOFA, sequential organ failure assessment; URTI, upper respiratory tract infection.

### Main findings

The geriatric population was a focus for this systematic review as it presents a unique perspective in addressing the treatment options for COVID-19. Despite the drastic impact that COVID-19 has had on this population with respect to morbidity and mortality rates, there is little research focusing on this population.^4^ Studies focusing on FVP, BMB, and BNB in the treatment of COVID-19 was found to be useful in the geriatric population. In contrast, HCQ, DMS, RMD, and anticoagulant therapy have shown inefficacy in the geriatric population, as studies revealed no change or increased mortality and had minimal to no clinical benefits. There is conflicting evidence on the utility of TCZ, CP, supportive therapy, and anticoagulant therapy as there were mixed results in supporting the efficacy on mortality, ventilator-free days, and clinical improvements in the geriatric population in the reviewed data. In addition, there was limited evidence and lack of data due to ongoing trials for treatments with SBV and BUD. A significant decrease in viral load and improvement in clinical symptoms subsequent to these modalities was observed in some of the studies reviewed in the geriatric population. However, a large number of these studies utilized a wide patient demographic (e.g. age ranges) making it difficult to conclude the exact physiological effects various treatments are expected to have on older patients (e.g. shortened duration of COVID-19 symptoms, clinical improvement, decreased viral load, decreased inflammatory markers).^10,13,17,22,24,27–29^

### HCQ treatment

Of the 14 studies focusing on HCQ, 6 studies showed positive changes in primary patient outcomes.^10,11–14,20^ Eight studies showed no statistically significant difference.^15–19,39–41^ Further to this, a large sample RCT noted that HCQ did not lower the incidence of death at 28 days compared with those that received usual care.^40^ It should be noted that in addition to HCQ in some of the reviewed studies, azithromycin (AZT) was co-administered to some patients.^11,13,17,18^ In addition, cardiac arrest was more frequent in patients who received HCQ with AZT compared with patients who received neither drug.^1^

Studies looking into the use of HCQ as a post-exposure prophylaxis have shown no significant prevention benefit in COVID-19.^20^ Boulware *et al.* (2020) demonstrated that individuals with a severe to moderate risk of developing COVID-19 based on previous exposure who were treated prophylactically with HCQ were at greater risk for side effects compared with a placebo group (40.1% to 16.8%).^61^

### Corticosteroids

#### DMS treatment

Two of the analyzed studies supported the use of DMS in the treatment of COVID-19. One study noted a significant decrease in mortality seen in patients treated with steroids compared with controls (13.9% *versus* 23.9%).^37,43^ Among patients with COVID-19 and moderate or severe ARDS, use of intravenous DMS plus standard care compared with standard care alone resulted in a statistically significant increase in the number of ventilator-free days (days alive and free of mechanical ventilation) over 28 days.^37^ In contrast, there was no significant change in mortality rate between steroid treated and control patients, and increased corticosteroid dosage was associated with significantly elevated mortality risk.^35^ Studies that examined other corticosteroids other than DMS such as hydrocortisone and methylprednisolone, showed a significant increase in mortality rates observed in treatment groups compared with control groups.^46,47^

#### BUD treatment

There was one large open-labeled RCT conducted examining BUD. Yu *et al.*^55^ examined 751 individuals aged  > 65 who were randomized to receive 800 µg of inhaled BUD, twice daily for 14 days. It was found that inhaled BUD reduced self-reported time to recovery from 11 to 2 days (*p* = 0.017), hospital admission (*p* = 0.81) or death estimated outcomes, and serious adverse events compared with standard COVID-19 care.^55^

### Antiviral treatment (RMD and FVP)

RMD was not found to be effective in improving clinical symptoms in patients in most studies reviewed in this systematic review.^43,62–64^ The findings from the Grein *et al.*^22^ study revealed that, in patients of 70 years of age and older, clinical improvement was observed less frequently than in patients less than 50 years of age [hazard ratio (HR) = 0.29 95% CI = 0.11–0.74]. The lack of a significant decrease in mortality with RMD treatment noted by Beigel *et al.* further supports the conclusion that RMD may not be as effective for COVID-19 treatment in the geriatric population.^21^

FVP has been proposed as a treatment for patients with COVID-19 and is supported in two of the three studies reviewed in this systematic review.^33,34^ There was an observed statistically significant decrease in duration to patient viral clearance (*p* < 0.001) and increased rates to clinical recovery (*p* < 0.05) from COVID-19-related symptoms (fever, dyspnea, decrease in oxygen saturation, cough) with the use of FVP.^33,34^ Furthermore, in previously untreated patients, FVP had a higher clinical recovery rate (recovery of fever, respiratory rate abnormalities/instability, oxygen saturation, cough).^34^

### Monoclonal antibodies (TCZ, BMB, BNB, SVB)

#### TCZ treatment

The existing limited body of literature provides mixed evidence for its use, with only half of the identified RCTs suggesting benefit to clinical outcomes and overall survival benefit.^44,49^ One study found the risk of in-hospital mortality was lower in patients treated with TCZ in the first 2 days of intensive care unit (ICU) admission compared with those that received alternate treatment. Furthermore, the estimated 30-day mortality in the intervention group was 27.5% compared with 37.1% in the non-intervention group.^44^ These findings were supported by a large-scale RCT comparing TCZ with standard of care (UC), where clinical benefit and survival outcome were observed more often in those treated with TCZ regardless of level of respiratory support and addition of systemic corticosteroids.^49^ Two additional RCTs did not observe a benefit to COVID-19 disease progression compared with UC; however, both studies were limited by small sample size and narrow patient demographic.^44,45^

#### BMB treatment

An RCT conducted by Gottlieb *et al.* found that treatment with BMB and etesevimab compared with placebo was associated with a statistically significant (*p* = 0.01) viral load reduction at day 11; however, there was no significant viral load reduction using BMB monotherapy. Immediate hypersensitivity reactions were reported for 9 out of 613 patients with BMB monotherapy, and no deaths occurred during the treatment study.^48^ An OCS examined the use of BMB in nursing facilities revealed a side-effect rate of 11.1% which included hospitalization and emergency department admissions with no deaths.^65^ Gottlieb *et al.* who examined BMB in a combination dose concluded that, it is unclear whether monotherapy or different combinations have more clinical efficacy.

#### BNB treatment

There were five novel studies found examining BNB in older patients with COVID-19. Abizanda *et al.*^49^ examined patients hospitalized with moderate-to-severe COVID with pneumonia; 328 patients were stratified to older or younger than 70 years old given 4 mg of BNB for 14 days. In the  >70-year-old group, treatment with BNB significantly reduced death from any cause (48%) and 30-day absolute mortality (20.5%) compared with controls without BNB (*p* < 0.001).

Stebbing *et al.* 2021 conducted an observational chart review in 122 patients who were given high-dose BNB (8 mg), compared with low-dose BNB (4 mg) for 14 days in older patients. In the high-dose BNB group, blood oxygen saturation was stabilized earlier (*p* < 0.05) and had reduced intensive support (*p* < 0.05), reduced 30-day mortality (*p* < 0.05) and 60-day rehospitalization rate (*p* < 0.01) compared with the low-dose BNB group.^66^

In Bronte *et al.*, a longitudinal observational study, 20 patients with COVID were using 5 mg of BNB twice daily for 2 days, followed by 5 mg per day for 7 days. Patients treated with BNB had a reduction of serum IL-6, IL-1B, and TNF-alpha, rapid recovery of circulating T- and B-cell frequencies and increased antibody production to the spike protein.^51^ In addition to the immunological findings, there was 1 death in the BNB group compared with 25 deaths in the group without BNB (*p* < 0.001), a decrease in ARDS (*p* = 0.37), with a similar duration of hospital stay for both groups.^51^

In Rosas *et al.*, a retrospective review on patients with confirmed COVID-19 comparing 23 patients who received BNB monotherapy (2–4 mg daily) and 31 patients who received TCZ (IV dose 400 mg). There was no significant mortality benefit or side effects noted with either treatment group; however, BNB monotherapy showed a significant reduction in temperature, CRP, D dimer, increase in oxygen saturation requirement, and decrease of respiratory rate compared with TCZ (*p* < 0.01), and less intensive care intubation (27% reduction).^52^

In Izumo *et al.*,^53^ an observational study of 44 patients with severe COVID-19 received triple therapy of BNB (4 mg daily oral), RMD (IV 200 mg loading, 100 mg maintenance daily) and DMS (oral or IV 6 mg daily). It was found that patients who received BNB had a low mortality rate (2.3%) and decrease in mechanical ventilation requirement (90%) with approximately 34% adverse events observed.^53^

#### SVB treatment

There was one multi-center RCT that had a geriatric cohort over 65 years of age, which randomized 93 patients into an SBV group (500 mg IV), and 93 who received placebo.^54^ Three patients in the SVB group, compared with 21 patients in the placebo group had disease progression leading to hospitalization or death (*p* = 0.02). In addition, adverse events were reported in 17% of the SVB patients compared with 19% in the placebo group.

### CP treatment

A decrease in viral load and an improvement in clinical symptoms of fever, dyspnea, cough, and chest pain was noted in reviewed studies in patients who received CP.^24,23,26,27–29^ Three RCTs that used CP as treatment did not report any significant difference in time to clinical improvement or overall mortality between patients who received CP therapy compared with the control group.^38,42,67^ Likewise, the RCT by Piechotta *et al.* supported the uncertainty of the use of CP in the effect on mortality and hospital discharge for COVID-19 patients.^68^

### Supportive therapy

Patients who were provided with a daily PP session in the reviewed studies showed decreased mean respiratory rate from 28.4 ± 3.5 breaths/min to 21.3 ± 1.3 breaths/min and an improvement in oxygen saturation during PP compared with baseline.^30,32^ However, Elharrar *et al.* reported that 42% of the patients who received this therapy experienced back pain due to the PP.31

### Anticoagulant therapy

There were five studies that met the inclusion criteria treating geriatric patients with anticoagulant therapy. Fumagalli *et al.* (2022), retrospective observational study (ROS), on patients aged  > 60 with atrial fibrillation who were on antiplatelet or oral anticoagulant therapy, found that oral anticoagulant use before (*p* < 0.001) and during hospitalization (*p* = 0.002) was higher among survivors.^56^ In addition, higher self-sufficiency, less severe initial COVID-19 presentation, and the use of vitamin K antagonists or direct oral anticoagulants (DOACs) on admission, or the persistence of DOAC during hospitalization were associated with a lower chance of in-hospital death.^56^

Rossi *et al.*, retrospective OCS for patients aged  >70 with COVID-19 and interstitial pneumonia with known coronary heart disease (CAD), who were on long-term anticoagulant or antiplatelet therapy including aspirin, P2Y12 inhibitors, dual antiplatelet therapy, DOACs, beta-blockers, statins, angiotensin converting enzymes (ACEs), angiotensin II receptor blockers (ARBs), or calcium-antagonists.^57^ Long-term anticoagulant use in the survivor group was higher than non-survivor group (*p* < 0.001), specifically DOACs were associated with decreased mortality (*p* < 0.01) compared with other anticoagulation or cardiac therapies.

Olcott *et al.*, a ROS on patients aged  >70 who were admitted with a confirmed COVID-19 infection, where 22% of patients were on DOACs, 4% were on warfarin and 74% were the comparator group on no anticoagulant therapy, found no statistically significant improvement in all-cause mortality for patients who were anticoagulated preadmission (*p* > 0.05).^58^

An RCT by Sadeghipour *et al.*, randomized 562 patients to receive intermediate-dose (*n* = 274; enoxaparin, 1 mg/kg daily) *versus* standard prophylactic anticoagulation (*n* = 286; enoxaparin, 40 mg daily), found that intermediate-dose prophylactic anticoagulation, compared with standard-dose prophylactic anticoagulation did not result in a significant difference in venous or arterial thrombosis or morality within 30 days (*p* = 0.70). Interestingly enough, severe thrombocytopenia was noted in the intermediate-dose, and not in standard-dose anticoagulation cohort (*p* = 0.01).^59^ Another RCT by Zarychanski *et al.* examined patients with severe COVID-19 on therapeutic anticoagulant therapy (unfractionated or low molecular weight heparin) *versus* thromboprophylaxis (DOAC), noted that therapeutic anticoagulation did not improve hospital survival (64.3% *versus* 65.3%) or days free of organ support (3 *versus* 5 days) compared with thromboprophylaxis.^60^

## Discussion

### HCQ use in the geriatric population

In the studies analyzed in this review, the consensus on HCQ as an effective treatment is variable. The studies that concluded positive antiviral effects of HCQ had smaller sample sizes compared with studies that concluded no significant antiviral effects of HCQ. HCQ was proposed as an effective treatment for patients with COVID-19 due to its antiviral, anti-inflammatory, and antithrombotic effects.^25^ However, potential side effects of HCQ need to be considered prior to starting geriatric patients on this drug. The therapeutic combination of HCQ with AZT may have influenced the primary outcomes in these studies. However, there was no agreed consensus between these studies that used combination HCQ/AZT therapy on an improvement or lack of improvement in terms of the primary outcomes.

As of 24 April 2020, the Food and Drug Administration has cautioned the use of HCQ outside of hospital due to the ill side effects of the drug-based RCT.^25^ In addition, clinical guidelines are not recommended HCQ due to lack of benefit, potential harm, and system implications of overuse.^69^ The use of adjunctive therapy seen in the studies in which AZT was co-administered with HCQ, may have didanosine (DDI), which could lead to a synergistic effect and impact the efficacy and outcomes of the primary treatment assessed in the studies.

The most commonly reported side effects of HCQ post-exposure prophylaxis included nausea, loose stools, and abdominal discomfort.^36^ QT prolongation to > 500 ms has also been observed with the use of HCQ and is a known marker for malignant arrhythmia and sudden cardiac death.^15,16^ HCQ and AZT combined have higher incidence of torsade de pointes, ventricular tachycardia, or cardiac arrest if taken (>4 days) and can result in cardiac-related mortality, which can be detrimental in older individuals with pre-existing heart conditions.^70,71^ When aggregating the results of HCQ, six studies showed positive effects and eight studies had negative side effects.

There was a high risk of bias in the HCQ studies (Table 2). High attrition bias was noted in some of the analyzed studies, particularly evident in the HCQ studies, due to early cessation of the treatment, death of patients, or lack of patient follow-up.^10,14,17,20,22^

**Table 2.** Risk of bias of analyzed studies.

	Selection bias – Sampling technique and allocation of treatment	Performance bias – Blinding of participants and personnel	Detection bias – Blinding of outcome assessment	Attrition bias – Incomplete outcome data of participants	Reporting bias – Selective reporting	Other bias
Ahmad *et al.*^10^	High	High	High	High	High	Not specified which patients and how many patients received the higher dosage of HCQ in the combination HCQ-Doxycycline treatment. No stratification or comparison based on the different dosages of HCQ in the treatment.
Carlucci *et al.*^11^	Low	Low	Mid	Low	High	No adjustment for the difference in timing between patients who did not receive zinc. The time of initiation of zinc may have differed since the diagnosis.
Chen *et al.*^12^	Low	Low	Low	Low	Low	All patients studied were from the same hospital and were admitted in the same month (Feb 2020).
Gautret *et al.*^13^	Low	High	High	High	Low	Patients outside the main study center were used as controls.
Gautret *et al.*^14^	High	High	High	Low	High	
Geleris *et al.*^15^	High	High	High	Low	Low	Missing data, and potential inaccuracies in the electronic health records. Single center design limits the generalization of the results.
Mahévas *et al.*^16^	High	High	High	N/A	Mid	Observational data of treatment were not randomly assigned, and potential cofounders could bias the results.
Molina *et al.*^17^	High	High	High	High	High	
Rosenberg *et al.*^18^	Low	Low	Low	Low	Low	Large random sample from 25 metropolitan hospitals. Significance was evaluated at α = .05 and all testing was two-sided. Interpretation limited by the observational design.
Tang *et al.*^19^	Low	High	Low	Low	Low	Open-label as opposed to double-blind design introduces the possibility of biased investigator determined assessments and unbalanced dosage adjustment.
Yu *et al.*^20^	High	Low	Low	High	Low	Single hospital study, patients recruited from 1 February 2020 to 8 April 2020. Disproportionate allocation of subjects to control and treatment groups.
Beigel *et al.*^21^	Low	Low	Low	Low	Low	Training, site initiation visits, and monitoring visits often were performed remotely due to implementation during a time of travel restrictions.
Grein *et al.*^22^	High	High	High	High	High	Patients observed were from multiple countries. Patients were diagnosed with severe CoV-19 and oxygen saturation of less than 94%.
Wang *et al.*^5^	Low	Low	Low	Low	Low	Restrictions on hospital bed availability resulted in patients enrolled in the study were later in the course of the disease.
Ahn *et al.*^23^	High	High	High	N/A	High	Low number of cases. Number of antibodies administered to each patient was not standardized.
Duan *et al.*^24^	High	High	High	N/A	High	Historic control group – not random.
Li *et al.*^25^	Low	High	High	Low	Mid	Sample size was small and study terminated early.
Liu *et al.*^26^	High	High	High	Low	Mid	Sample size was small and not stratified into subgroups. Patients received additional medications (anticoagulants) that may have confounded clinical outcomes.
Salazar *et al.*^27^	Low	High	High	Low	Low	Limited sample size – no control. Patients treated with other medications (HCQ, AZT, RMD, TCZ, and methylprednisolone).
Shen *et al.*^28^	High	High	High	N/A	High	Limited sample size – not random.
Ye *et al.*^29^	High	High	High	N/A	High	Limited sample size – not random.
Dong *et al.*^30^	High	High	High	High	High	Duration of PP or LP positioning varied between patients. Standard treatment of antivirals, antibiotics, anticoagulation, and nutritional support was given to patients when required.
Elharrar *et al.*^31^	High	High	High	N/A	Mid	Sample size was small. A single episode of PP was evaluated; follow-up was short, clinical outcomes were not assessed, and causality of the observed changes cannot be inferred.
Sartini *et al.*^32^	High	High	High	N/A	High	Small number of patients, short duration of non-invasive ventilation in the PP, and lack of a control group. Selection bias highly possible. Patients in the study may not be representative of all patients with CoV-19 treated with non-invasive ventilation in the PP.
Cai *et al.*^33^	High	High	High	Low	Low	Relationship between viral titers and clinical prognosis was not well clarified.
Chen *et al.*^34^	Low	High	High	Mid	Low	Endpoint duration varied between patients. Imbalance of proportion of critically ill patients between groups.
Lou *et al.*^35^	High	High	High	Mid	High	Small sample size. Dosing varied between patients within the same treatment group. All patients continued to receive existing antiviral treatment in addition to experimental protocol. No statistical analysis performed.
Lou *et al.*^35^	Low	High	High	Low	High	All patients received antiviral treatment. Duration of steroid treatment varied between patients in treatment group. Did not take into account patients who currently receive steroid treatment for comorbid conditions (e.g. chronic obstructive pulmonary disease).
Wu *et al.*^36^	Low	High	High	Low	High	Variable treatment dosing and types of steroids used.
Tomazini *et al.*^37^	Low	High	High	Low	High	35% of the patients in the control group received corticosteroids during the study period, possibly related to the open-label design, the disease severity of the patients, and other diverse indications for corticosteroid use in critical care.
Simonovich *et al.*^38^	Low	Low	Low	Low	Low	Although the use of usual therapy was allowed in both groups, it was not standardized among participating sites.
Mitja *et al.*^39^	Low	High	High	High	Low	
RECOVERY Collaborative Group^40^	Low	Low	High	Low	Low	
Self *et al.*^41^	Low	Low	Low	Low	Low	
Agarwal *et al.*^42^	Low	Low	High	Low	High	Study used an open-label design, and was susceptible to anchoring bias of the treating doctors in outcome ascertainment.
Goldman *et al.*^43^	Low	High	High	High	Low	
Gupta *et al.*^44^	Low	High	High	N/A	High	Treatment groups differed at baseline before applying inverse probability weighted, with TCZ-treated patients being younger and having fewer co-morbidities, but also being more likely to have hypoxemia and elevated markers of inflammation, compared with non-TCZ group.
Salvarani *et al.*^45^	Low	Low	Low	Low	Low	
Hermine *et al.*^46^	Low	Low	Low	High	Low	Trial targeted a narrow segment of the COVID-19 patient population (patients with a World Health Organization-Combined and Positive score of 5 and requiring at least 3 l/min oxygen). These results were not generalizable to other populations.
Horby *et al.*^47^	Low	Low	Low	N/A	Low	Following random assignment, 17% of patients in the TCZ group did not receive the treatment. For reasons not recorded.
Abizanda *et al.*^49^	High	High	High	Low	Low	
Stebbing *et al.*^50^	High	High	High	Low	Low	
Bronte *et al.*^51^	High	High	High	High	Low	Small sample size (20 patients). Missing outcome data for immunological parameters.
Rosas *et al.*^52^	High	High	High	Low	Low	Small sample size (60 patients, only, 23 received BNB)
Izumo *et al.*^53^	High	High	High	Low	Low	No comparison group, every patient received triple therapy of BNB, RMD, and DMS.
Gupta *et al.*^54^	Low	Low	High	Low	Low	
Yu *et al.*^55^	Low	Low	Low	Low	Low	Limited data for hospitalization and death.
Fumagalli *et al.*^56^	Low	High	High	Low	Low	
Rossi *et al.*^57^	Low	High	High	Low	Low	
Olcott *et al.*^58^	Low	High	High	High	Low	Authors only had inpatient data of prescribed anticoagulants in hospital. There was a risk that individuals had primary care prescription of anticoagulants prior to admission which were not recorded.
Sadeghipour *et al.*^59^	Low	High	Low	Low	Low	No double blinding (open-label).
Zarychanski *et al.*^60^	Low	High	Low	Low	Low	No double blinding (open-label).

AZT, azithromycin; BNB, baricitinib; DMS, dexamethasone; HCQ, hydrochloroquine; LP, lateral position; PP, prone position; RMD, remdesivir; TCZ, tocilizumab.

### Corticosteroid use in the geriatric population

#### DMS

Corticosteroids, including DMS and BUD are immunomodulators that may act to suppress the inflammatory storm, reduce inflammatory exudation, and prevent multiple organ injuries in the context of COVID-19 ARDS.^72^ Studies that examined hydrocortisone and methylprednisolone compared with DMS showed a significant increase in mortality rates observed in treatment groups compared with control groups possibly due to suppression of the immune response that allows for virus replication.^62,72^ The immunosuppressant nature of systemic corticosteroids leads to reduced lymphocyte count which may lead to higher risk of superinfections.^72^ Clinical guidelines recommend DMS only for moderately and critically ill patients requiring low-flow supplemental oxygen and ventilatory support; however, it is not recommended for mildly ill patients.^69^

The risk of bias in the studies for DMS was high due to the lack of standardization of the treatment doses (i.e. 6 mg, 10 mg, 20 mg, or not reported route of administration; for example, oral compared with intravenous), and duration of treatment (e.g. 5 days, 10 days, or until ICU discharge) which varied between patients within the same study, and between all the analyzed studies (Table 2).^35,37^

#### BUD

RCT that examined BUD in the geriatric population revealed positive outcomes in recovery time, hospital admission, and death estimated outcomes.^55^ This is consistent with current clinical guidelines, and inhaled BUD may be considered especially for symptomatic high-risk immunocompromised patients who are mildly ill and not requiring supplemental oxygen.^69^

Even though there was only one large multi-center RCT examining BUD, the risk of bias was low as the treatment dose was standardized and it was as a large multi-center RCT (Table 2).^55^

### Antiviral (FVP and RMD) in the geriatric population

#### FVP

As of 22 October 2020, the US Food and Drug Administration granted approval for RMD as treatment of COVID-19 among patients requiring hospitalization.^36^ Similarly on 28 July 2020, the Health Canada authorized RMD as a treatment of patients with severe COVID-19.^21^ Current clinical guidelines recommend RMD for moderately ill patients who are newly requiring low-flow supplemental oxygen; however, it is not recommended for critical ill patients receiving mechanical ventilation.^69^ However, despite the proposed antiviral mechanism of action of RMD of impairing viral RNA production by obscuring viral RNA polymerase and inhibiting viral exonuclease, RMD was not found to be effective in improving clinical symptoms in patients in most studies reviewed in this systematic review.

FVP is a nucleoside analogue antiviral drug with a similar mechanism of action to RMD but is taken orally. FVP has been used in the treatment for many types of influenza virus strains, Ebola, arenavirus, bunyavirus, filovirus, West Nile virus, yellow fever virus, Coxsackievirus, and Lassa virus.^18^ Furthermore, the control treatments that FVP was compared against when drawing conclusions on its antiviral activity were different between the studies. Of particular consideration is the variability in dosing between patients of the same treatment group which may have largely affected the conclusions drawn by this study, which was the only study to conclude that FVP did not provide any further benefit over the control treatment.^35^

The exact dosing of FVP and duration of treatment varied largely between the analyzed studies, notably dosing of 600 mg twice daily to 1600 mg twice daily and duration of 10–14 days. An advantage of FVP in patient treatment for COVID-19 is its oral administration, thus allowing it to be easier utilized in an outpatient setting to symptomatic patients.^35^

The risk of bias in these studies was low due to well-constructed design and statistical significance of the reported data.^62–64,43^ It should be noted that blinding was not performed in any of the analyzed studies focusing on FVP which may have given a possible source for performance and detection bias.^33,48^

#### RMD

RMD was not found to be effective in improving clinical symptoms in patients in most studies reviewed in this systematic review.^62–64,43^ Despite that the current clinical guidelines recommend RMD for moderately ill patients requiring low-flow supplemental oxygen, it is not recommended for critically ill patients requiring ventilatory support or mildly ill patients.^69^ This suggests that RMD should not be used as firstline in geriatric patients and can be considered only in moderately ill population requiring minimal supplemental oxygen.

The risk of bias was low in half of the RMD RCT studies that were blinded.^5,21,43^ However, two OCS studies had a small sample size, and no control group or blinding performed, which may lead to a possible performance and detection bias.^27,53^

#### TCZ

TCZ is a recombinant humanized anti-IL-6 receptor monoclonal antibody that inhibits the binding of IL-6 and soluble IL-6 receptors to membrane, blocking IL-6 signaling and thereby reducing inflammation.^44^ It is commonly used in the treatment of rheumatoid arthritis, and most recently considered for use in adult patients, with an age range of 52–71, hospitalized with severe COVID-19.^44,49^

There were conflicting outcomes for TCZ in the review. In critically ill patents  >60 years old, TCZ has shown benefit for lowering the risk of death and faster hospital discharge.^44,49^ In other studies, it showed no benefit on disease progression.^45,51^ In addition, compared with other monoclonal antibodies such as BNB, TCZ had worsened improvement in respiratory rate and in oxygen saturation.^67^ Despite this conflicting evidence, TCZ is currently recommended for moderately and critically ill patients with serum CRP of 75 mg/l or higher and evidence of disease progression based on respiratory or ventilatory parameters.^69^

The risk of bias of the TCZ studies were high in one of the observation studies, with limited blinding of participants, outcomes, and selective reporting results from younger patients (<60).^65^ The three other RCTs had an adequate blinding and randomization which minimized the performance and detection bias.^49,51,66^

#### BMB

Novel treatments for COVID-19 have been geared toward biologics, specifically monoclonal antibodies. BMB is a neutralizing IgG1 monoclonal antibody that works against the SARS-CoV-2 spike protein.^48,65^ BMB has been used both as a monotherapy and a combination therapy with etesevimab or casirivimab-imedevimab, which have the same antibody properties as BMB. It was concluded that BNB monoclonal therapy was generally well tolerated and beneficial for the geriatric population.^65^

Early data on monoclonal antibody treatment suggest that high-risk patients including those older than 65 years may benefit from BMB and other approved biologics.^73^ The National Institutes of Health (NIH) has only approved BMB for mild-moderate symptoms of COVID-19 in older adults.^74^ It is unclear whether BMB can be beneficial for the geriatric population with severe symptoms. Currently, BMB is not recommended based on the clinical guidelines.^69^

It should be noted that blinding was not performed on the single BMB study, which may have resulted in performance and detection bias.^48^ Furthermore, patient population was small, limiting generalizability.^48^

#### BNB

BNB, a Janus kinase (JAK) inhibitor, originally approved for rheumatoid arthritis, has been approved for emergency use authorization by the Food and Drug Administration.^75^ JAK inhibitors can prevent proinflammatory cytokines and have shown clinical usefulness in inflammatory diseases, and thus has been evaluated for treatment of COVID-19.^76^ Current clinical guidelines recommend the consideration of BNB for moderately ill patients on low-flow supplemental oxygen and for critically ill patients requiring ventilatory support who are also on DMS or have contraindications to corticosteroid therapy.^69^

Although there is increasing evidence for the use of BNB in the older population, all of the studies reviewed were observational. These studies have the high selection and performance bias, as there was no blinding of participants, personnel or outcome assessment. Furthermore, known and unknown confounding variables could have affected the reported results as well. In addition, three of the five reviewed studies had small sample sizes limiting overall statistical power.^51–53^ The study by Bronte *et al.* has missing data for outcomes of immunological parameters which may have skewed the results and did not allow statistical power calculations for the flow cytometry results. The last study conducted by Izumo *et al.* was conducted at the Red Cross, which had an environment that differed from traditional hospital resources and could have other factors contributing to the reported results.^53^

#### SVB

The WHO group has made a conditional recommendation for the use of SVB in patients with non-severe-COVID but at high risk of hospitalization.^77^ Current clinical guidelines only recommend SVB for patients who are mildly ill, presenting within 7 days of symptom onset, which may include residents of long-term care facilities, inpatients with nosocomial infection, and high-risk patients  >70 years of age with comorbidities such as diabetes, cerebral palsy, sickle cell disease, or immunocompromised.

As the single SVB study was a large multi-center double-blinded RCT and the dose was standardized to 500 mg of SVB for each patient, there was minimal risk of bias, but there were only three patients in the SVB group who were hospitalized after treatment, which led to difficulties in determining which patient characteristics were associated with treatment failure.^54^ A major limitation of this study was lack of reporting of secondary outcomes such as the percentage of patients with emergency visits and the need for supplemental oxygen, as the trial is still ongoing. A recent study has shown benefit of SVB treatment in patients without immunity to COVID-19 (vaccine or disease-induced) within early onset of symptoms (<7 days).^54^ This early use of SVB is reflected in current clinical guidelines which recommend against SVB in moderately ill patients requiring supplemental oxygen and critically ill patients requiring ventilatory support; however, SVB may be considered in mildly ill patients who do not require new or additional supplemental oxygen from their baseline.^69^

### CP in the geriatric population

Immunomodulation through the passive transfusion of CP to patients acts to add neutralizing antibodies, anti-inflammatory cytokines, clotting factors, and defensins to the patient’s blood to enhance the immune system’s ability to clear the virus. This may be effective treatment for COVID-19, as it is associated with prevention of the over-activation of the immune system, and as a result, the ‘cytokine storm’ which may perpetuate pulmonary damage seen in infected patients.^78^ However, current clinical guidelines do not recommend CP for COVID-19.^69^

There is a high risk of bias in CP studies. All the studies included in the review lack significance of the results and as a result, it is difficult to draw conclusions.^10,23,24,27–30^

### Supportive therapy in the geriatric population

When considering supportive therapies for older individuals, it is important to consider the possible harms that therapy may have; PP was reviewed in the assessed studies, and while PP may have a supportive role in COVID-19 patients, it may exacerbate or contribute to back pain. The physical pain experienced by these patients may have affected the duration that they could undergo PP and subsequently the efficacy of this therapy.^30–32^

PP and LP studies have high risk of bias, as all studies were observation and were not standardized by patient’s position. The mean age of the included population was 54.4 years with no comorbidities. The treatment was administered concurrently with other interventions such as antibiotics, anticoagulants, and antiviral medications.^30^ In addition, the sample size was small in multiple studies, with lack of a control group, which led to increased risk of selection bias.^31,32^

Other supportive interventions that did not meet the inclusion criteria of this review may be helpful for COVID-19 patients, and balance should be found between the potential benefits and adverse effects. Lorazepam has been used in the management of anxiety in patients with COVID-19.^79^ However, benzodiazepines may exacerbate mental status changes, cause increased sedation, and lead to the development of delirium in this population.^80^ Opioids and oral hydration have been used to alleviate pain, dyspnea, and keep patients comfortable in palliative care settings.^79^ Oxygen functional materials (OFM) used to deliver oxygen therapy to palliative geriatric patients may impair the interaction and communication between the patient and their family, lead to xerostomia, and need for intravenous hydration, which may have a negative impact on quality of life, increase pulmonary secretions and generalized edema.^81^ Studies have illustrated the potential for traditional Chinese medicine as an effective treatment for COVID-19 and improvement of clinical symptoms.^82,83^ Additional supportive strategies, in addition to PP, should be considered if the benefits of treatment outweigh the adverse effects to the older patient and should be based on patient goals of care. Furthermore, these supportive strategies may be used synergistically to any upcoming accepted treatment for COVID-19.

### Anticoagulant therapy in the geriatric population

Prophylactic dose of anticoagulation is recommended in moderately ill patients requiring low-flow supplemental oxygen, whereas therapeutic dose anticoagulation may be considered for those who are at low risk for bleeding.^71^ However, a therapeutic dose of anticoagulant therapy is not recommended for critically ill patients, instead a prophylactic dose specifically of low molecular or unfractionated heparin is recommended.^69^ These clinical guidelines are for adults, and not necessarily tailored toward patients  >65 years of age with comorbidities that put them at a higher baseline of bleeding.

Of the five studies that met inclusion criteria, three had negative or negligible effects in the geriatric population with severe COVID-19 requiring hospitalization, two of which were large RCTs that revealed that neither prophylactic nor therapeutic anticoagulation protect against venous or arterial thrombosis, mortality, or days free of organ support in severe cases of COVID-19.^59,60^ This does not support the current clinical guidelines, as both therapeutic and prophylactic anticoagulation therapy can even lead to severe thrombocytopenia^79^ and major bleeding in COVID-19 patients.^60^ Two of the studies supporting anticoagulant therapy were observational studies assessing moderate COVID-19 cases and had cohort with specific cardiac comorbidities such as atrial fibrillation or CAD.^56,57^

Overall, the risk of bias for the anticoagulant studies was high. Three of the observational studies were lacking blinding and randomization which may have led to performance and detection bias.^56–58^ A major bias in these retrospective reviews was incomplete outcome data from participants, as drug history was taken from pervious discharge letters and as a result, some patients may have commenced anticoagulation therapy out of hospital, in primary or secondary care which was not documented. In addition, both RCTs were open-label, without a double blinding for anticoagulant cohort allocation, and as such a drawn conclusion could be questioned.

### Additional COVID-19 treatments

Despite current clinical guidelines recommend FLV for moderately and critically ill patients, this treatment was not found in the reviewed data for the geriatric population based on the inclusion criteria. FLV is a selective-serotonin receptor inhibitor which may be considered for mildly ill patients with COVID, based on low certainty evidence of reduction in hospitalization, but the reviewed studies were lacking efficacy and safety against the new COVID-19 Omicron variant.^69^ As a result, FLV therapy should be used with caution in this cohort as there is a lack of evidence. Current guidelines for the NIH and the Centers for Disease Control and Prevention (CDC) assert that no agent is known to be effective for preventing COVID-19 after exposure to the virus.^74^

### Limitations

A lack of statistical reporting, single-centered studies, lack of blinding, selective reporting, and attrition bias due to voluntary withdrawal or death led to challenges in assessment of the reported outcome and the validity of the reviewed studies (Table 2). Further to this, some studies changed primary outcome measure after initiation of study protocol, leading to possibility of outcome bias.

The short follow-up and small sample size of some of these studies presents a challenge in determining the accuracy of the statistical significance of the results and its impact on the population of interest.

A major problem of these studies includes early cessation of treatment, death of patients, and lack of patient follow-up, as seen in HCQ studies.^10,12,14,15,22^ In addition, most of the HCQ studies were observational, which increased the risk of confounding factors that may have affected clinical outcomes. Furthermore, positive clinical outcomes for HCQ studies came from the observational studies, with small sample sizes.

Overall, there was minimal research conducted for corticosteroid use in the geriatric population. Out of the two DMS studies and one BUD study, there were a lack of standardized dosing (ranged from 6–20 mg, inhaled BUS and intravenously administrated DMS) and duration of treatment (ranged from 5 days to discharge) varying between all participants.^35,37,55^

There were unique limitations to both types of antiviral therapy. Two of the three FVP studies were RCTs; however, they were limited by small sample size^55^ and measured by viral clearance and imaging^33^ instead of clinical improvements. This limits the generalizability of FVP to the entire geriatric population. For RMD, the only clinically positive study was an observational study,^22^ the other RCTs found no significant decrease in mortality and no significant clinical benefits.^5,21^ This conflicting evidence limits the conclusion that RMD is safe and effective for the geriatric population.

Monoclonal antibodies also had a variety of limitations. Many patients in the TCZ groups had severe COVID and were in the ICU.^49,51,65,66^ The efficacy of TCZ on mild or moderate COVID severity in the geriatric population is unclear. There was only one large RCT for BMB; however, it was not blinded, and all patients were not  > 60 years old, limiting the generalizability of the reported outcome to the geriatric population. Similarly, there was only a single RCT for SVB with lack of reported secondary outcomes, as the follow-up trial is still ongoing.^26^ BNB studies had small sample sizes limiting their statistical power.^51–53^ In addition, all these studies were observational which may have had additional confounding factors.

Many of the analyzed studies for CP had less than 50 patients, which resulted in being under powered.^14,17,23,24,26,28,29^,^30,32^ Four of the seven studies were DOS without drawn conclusions and based only on individual patient observations.^23,28,29,81,68^ The study designs, reported outcome, and lack of statistical significance of the analyzed studies makes it difficult to assess the true effectiveness of CP in treatment for COVID-19 in the geriatric cohort. Each of the reviewed studies focusing on PP were statistically under powered as had less than 50 patients receiving the intervention. As a result, the reliability of these studies was questioned in this specific population.

The only supportive therapies found in this review included PP and LP, but these studies were observational, with small sample sizes (<25 patients) and were lacking standardized approach to positioning.^30–32^ In addition, the true efficacy of PP and LP is unknown, as other treatments such as antibiotics, anticoagulants, and antiviral medications were used at the same time.^30–32^ The efficacy of other supportive therapies such as antipyretics, opioids, antimicrobial agents, and oxygen are unknown.

Many anticoagulant studies were ROS with the exception of the two RCTs, which were not blinded.^56–60^ In addition, the retrospective analyses only had inpatient data, and not previous drug history outside of the hospital, which may have skewed the results.^58^

There is a lack of reported impact of age to outcome on the treatment. It is possible that the older patients did not respond well to the treatment, and therefore the effects of the treatment on non-geriatric patients may have impacted the studies’ conclusions on effectiveness of treatment. In addition, the studies that provided age demographic information as mean ± SD and median ± SD were lacking the exact age of the participants and therefore did not solely include individuals  >60 years old.

There was no standardized approach to the treatment in reviewed studies, and exact dosing and duration of treatments varied within each of the reviewed treatment modality. These differences may have influenced the outcome of the treatments and confounded the results. In some of these studies, adjuvants were administered to patients in addition to the specified main treatment modality, which may lead to bias in the reported outcome. The design in some of these studies (e.g. DOS and OCS) led to low levels of evidence and reduced power from which conclusions were drawn on the effectiveness of each of the reviewed treatments for geriatric patients infected with COVID-19. In addition, many of these trials are still ongoing, and data were not fully disclosed, awaiting peer review and future publication.^15,51,64^

### Future directions

The limitations of the analyzed studies may spark the need for further testing each of the reviewed treatments to determine the true accuracy in clearing the viral load and impact to clinical symptoms of COVID-19. Attention to studies employing isolated single treatment modality would be pertinent to reduce the confounding effect of DDI, particularly seen in studies exploring concomitant use of HCQ and AZT. Large multi-centered, double-blinded RCT specifically looked at older, age 60 and above, with standardized control group, and longer follow-up should be conducted to assess the most effective treatment for COVID-19 and to determine the effects each of the above treatment on mortality rates in COVID-19 patients as well as assess its efficacy, in order to minimize bias. There is a need for standardized reporting of the inclusion criteria, administered treatment, and minimum effective dose used to yield patient recovery, as well as outcome endpoints, in order to compare results in the geriatric population which will most likely differ from adults.

Further stratification of this population could be analyzed by comorbidities such as cardiovascular disease, DM1, HTN, chronic respiratory disease, cancer, and other immunocompromised conditions which are shown to increase mortality in this group age. From a health prevention standpoint, future RCTs should investigate the use of pre- and post-exposure prophylaxis for high-risk older patients.

Specifically, anticoagulant therapy studies suggest that there may be clinical efficacy in geriatric patients with heart disease and severe cases of COVID-19, not necessarily in patients with mild cases or other comorbidities which should be further explored and applied to COVID-19 severity.^59,60^ The role of FLV should be further investigated, as there is a lack of up-to-date evidence, and the existing challenges with drug-drug interactions poses a barrier for its utility in the geriatric population.

COVID-19 is a rapidly developing area; there is no known agent that is effective for preventing COVID-19 after exposure to the virus at this time; as a result, the gold standard of pharmacological treatment is constantly changing. Future consideration into biologics such as antibody treatment is warranted in the older adults.

## Conclusion

This review revealed that no agent is known to be effective for preventing COVID-19 after exposure to the virus and such, further research is needed to focus on older patients to ensure safety and efficacy of any of the reviewed above treatment protocols, in both pre- and post-exposure settings.
